# The Metropolitan Hospital Sunday Fund

**Published:** 1911-08-05

**Authors:** 


### THE METROPOLITAN HOSPITAL SUNDAY FUND.

### MEETING OF COMMITTEE OF DISTRIBUTION.
### HOW THE INSURANCE BILL MAY AFFECT THE HOSPITALS.
### FULL LIST OF GRANTS.

The Committee of Distribution of this Fund met at the Mansion House, under the Presidency of its Treasurer, the Rt. Hon. Sir T. Vezey Strong, P.C., on Tuesday, August 1. The members present included Sir William Church, Sir Savile Crossley, Sir Henry Burdett, Sir John Bell, Mr. F. H. Norman, Sir H. Pearce Gould, and Archdeacon Sinclair.

### The late Chief Rabbi.

The minutes of the last meeting having been approved and signed, the Lord Mayor reminded the meeting that this was the thirty-ninth anniversary distribution of the Fund. Since the Council last met it had suffered a severe loss by the death of the Chief Rabbi, Dr. Hermann Adler, who had devoted his time and energy to the Fund from its inception throughout its course; and he was sure the Council would agree that that loss had been very great indeed. He proposed the following resolution: "That the Council desire to express to the family of the Very Reverend Dr. Hermann Adler, the late Chief Rabbi, their very sincere sympathy in the loss which they, as well as this Fund, have sustained. The Council will long remember with gratitude the great services rendered to the Fund by Dr. Adler during the twenty-one years in which they had the advantage of his services and assistance."

Sir Savile Crossley seconded, and it was agreed to.

### A Successful Year.

The Chairman, continuing, said the year in respect of which the distribution was about to be made was one possessing many features of advantage, in that the Fund had received the energetic and enthusiastic help of clergymen and ministers of all denominations in pursuing its work. Yet, owing to circumstances which it was difficult to estimate correctly, it was matter for lament that the collections had fallen off in amount. Perhaps this year, more than in previous years, people had been away from London, and had missed attending their ordinary place of worship on the date fixed for the collection, and had forgotten the advice which was tendered to them, to send their contribution to their clergyman, so that the Fund should not suffer loss. That advice, he feared, had not been acted upon to the full, therefore the Fund was found to be some £6,000 down in its collections. While expressing great gratitude for the contributions to the Fund, he suggested that where clergymen knew that certain members of their congregation had been away on the occasion, and had not sent anything to the Fund, it would be well to send a private reminder to them, and so bring the amount of the Fund to at least what it was before. The suggestion was made with great submission, because it was felt that local circumstances must be taken into account, as well as others of which he had no knowledge.

### The Report Adopted.

Sir William Church proposed: "That the Report of the Committee of Distribution for the year 1911 be, and is hereby received, and that the awards thereby recommended be paid as soon as possible." In seconding the observations of the Lord Mayor concerning the Fund, Sir William reminded the Committee that the Fund had had a windfall this year, which it would not have in the future, namely, £8,000, the balance of the income available from Mr. Herring's estate, and that was joined to the estate previously received, bringing its total to over £24,000.

Mr. F. H. Norman seconded, and called attention to the fact that hospitals required more money than ever owing to the new scientific appliances needed, and the extent to which they were availing themselves of the resources of science. In addition, nursing was a more expensive matter, as nurses nowadays were drawn from a superior class compared to those in bygone days, and their standard of living was higher. And the Insurance scheme now being discussed was likely to be an additional source of expense. Every effort should be made to prevent the income of the Fund being diminished.

The resolution was unanimously carried.

### Why the Contributions have Decreased.

Rev. W. S. Swayne moved "That the thanks of the Council be, and are hereby, accorded to the Committee of Distribution for the care they have bestowed in the preparation of the awards." He expressed his satisfaction at the way in which the awards had been made, and cordially agreed with the remarks as to the desirability of keeping up the total of the Fund. But he feared that the next year or two were likely to be very difficult ones, and too much must not be expected from the sending of private letters, such as had been suggested, as that had already been done in some of the parishes which responded largely to the annual appeals. He believed the decrease was due to a variety of causes, one of which was the transfer of many people from the centre, such as Kensington, to the suburbs, so that in the latter there were not many small houses available, whereas more centrally situated districts had a large proportion of the houses empty. Again, the people had often proved themselves incapable of thinking much upon more than one subject at a time—and this year it was the Coronation—a regrettable state of things, but, he believed, a fact.

Sir A. Pearce Gould seconded, and, while heartily thanking those who made the appeals, paid a warm tribute to the gentlemen who sat round the table and carried out its arduous work. No one interested in or connected with hospitals could have anything but anxiety in the immediate future in reference to the Insurance Bill, and many subscribers to the Middlesex Hospital had intimated that they could not in future continue the same amounts to the hospital. He feared such a feeling would become extensive. Such a danger made it all the more imperative that a fund such as this, which appealed to other motives than mere philanthropy, should be maintained at a high level. The appeal which was made from the various pulpits should have equal force whether there was an Insurance Bill or not, and he therefore anticipated that the Fund would fill an even larger place in financing the hospitals of the great Metropolis.

### The Insurance Bill and Hospital Revenues.

Sir Henry Burdett moved: "That the thanks of the Council be and are hereby given to the editors of newspapers who have pleaded the needs of the hospitals and advocated the cause of the Fund." He said that morning's papers bore very good evidence of the indebtedness of the hospitals to newspapers, for they contained very full reports of the Committee discussion on the Insurance Bill in Parliament. In that discussion the Chancellor of the Exchequer took up the point that the hospitals would not suffer, but would gain by the Bill. But he (the speaker) believed that the reasons and premisses which the Chancellor gave rested upon misapprehension, and he thought some of the amendments moved in favour of the hospitals were not well advised. They contained a proposal that out of the insurance money which was to come to the sick man, one-half or some other proportion was to go to the hospital if he went in as an in-patient. But hospitals had consistently worked from the standpoint of always helping the sick man, and the worst of the cases were those who had many dependents upon them. In fact, the voluntary hospital was established for the relief of those very people who, in the hour of need, were unable to maintain themselves and their families in comfort. Therefore, while thanking those members of the House of Commons who brought forward those amendments, it should be understood that those connected with hospitals could not support them. He looked with favour upon the general principle of the Insurance Bill, because it was going to do an enormous good and give long-deferred justice to the humblest people; but when inquiring how the Bill was going to affect voluntary hospitals, one got on to quite another basis. It was not the hospitals which were going to do anything unasked. What had to be done was to meet the demands for hospital care which would be created by the passing of the Bill. And as the Bill provided the money for one group of cases in sanatoria, it was certain that if the State was to come to the voluntary hospital, as it would come, and require in-patient care for an increasing number of insured people, provision must be made to put voluntary hospitals on equal terms with sanatoria by taking power, under Clause 15, to authorise the Health Committees to make arrangements with the hospital authorities for that accommodation. That would simply mean a *pro-rata* payment for actual benefit given to the assured at the instance of the State. This could readily be made available in our hospitals by a slight readjustment of some portions of the buildings, and it would leave the voluntary principle in connection with hospitals quite untouched. If a grant or payment from the State meant an interference with the management, there was no precedent for it. What the hospitals could not object to—in fact, he thought they would welcome it—was that where payments *pro rata* were made, inspection should be made by a representative of the State. Mr. Lloyd George said, on the previous night, that there would not be any large diminution in the income to the hospitals, because in the Employers' Liability Bill—especially instancing collieries—there was no falling off, though there was expected to be, in the employers' subscriptions. He (Sir Henry) believed the Chancellor had been misinformed even on that point. But collieries belonged to the class of large employers, whereas the hospitals depended largely upon the smaller employers of labour; the bulk of hospital subscription lists were made up of small employers. Those employers would have a heavy charge under the Bill, and would not be able to afford the gifts they formerly gave. The Chancellor and some of the Labour members made a point that subscriptions should be given by approved societies under the Bill. But Mr. Lawson at once answered that at the London Hospital the whole of the contributions received in one year from approved societies was £50. Moreover, under the Bill, with the changes which would be introduced into the financial arrangements, it would not be possible for a well-managed hospital to accept a subscription from an approved society. Where a workmen's society or a friendly society gave £20, even if there were 800 or 1,000 members, each of them, owing to the impression which had been produced on their minds about voluntary hospitals, would feel that they were entitled during the year to have free all in-patient relief of every kind, however expensive it might prove to be. The help given to the insured must be upon a business basis, and that must be arrived at by placing hospitals on the same footing as sanatoria, which a very slight amendment of Clause 15 would effect. Mr. Austen Chamberlain believed there would be a loss in the Birmingham hospitals of 50 per cent. He (Sir Henry) had worked out the figures for the whole country, and came to the figure of 45 per cent. of the affected income, and in Scotland it was even higher. In London the case was different, as only 23 per cent. of the income for hospitals in the Metropolis could be affected by the Bill.

He concluded by paying a warm tribute to the generosity of editors of the London Press for their advocacy of the cause of hospitals, even when the pressure upon their space was, as in the present year, unprecedented.

The resolution was seconded by the Rev. E. H. Pearce, M.A., and carried unanimously.

### The Policy of Wait and See.

The Hon. Sidney Holland remarked that when a deputation, of which he was a member, waited upon the Chancellor of the Exchequer, that gentleman asked a question which no member of the deputation could answer, namely, Why should working men subscribe less to hospitals under this Bill? They would be better off, because until now for 6d. they had received certain benefits from their insurance company which in future they would get for 4d. He (Mr. Holland) suggested that people suffered more from what did not happen than from what did, and he counselled that they should wait and see what would happen. Subscriptions did not fall off after the passing of the Employers' Liability Bill, nor after the Workmen's Compensation Bill; they did after the Licensing Bill, but only among brewers.

Sir Henry Burdett replied that he could only repeat that a large proportion of subscriptions were from workpeople, and if they were compelled to pay towards Government insurance they were less likely to subscribe to hospitals.

Sir Savile Crossley agreed that if workmen were making compulsory payments, they were less likely to make voluntary ones.

Sir John Bell proposed: "That the cordial thanks of the Council be and are hereby given to the Right Hon. Sir Vezey Strong, P.C., etc., Lord Mayor, who, as President and Treasurer, has devoted his valuable time and influence to the well-being of the Fund." He supported it with some warm and graceful words of appreciation, and it was carried with acclamation.

The Lord Mayor, in acknowledging the vote, reminded the meeting that nearly £2,000,000 had been distributed by the Fund to the hospitals of London, and for that he expressed the great satisfaction he felt, and said he hoped the benevolence of the people would enable that great Fund to accomplish still more in the future.

### REPORT OF THE COMMITTEE OF DISTRIBUTION TO THE COUNCIL.

The Committee of Distribution held their first meeting on Tuesday, May 23, and then and at subsequent meetings examined the reports and income and expenditure accounts of the several institutions seeking to participate, and made their awards in conformity with Law V.

The executors of the late Mr. George Herring will pay to the Treasurer of this Fund on July 28, £8,000, being the balance of income available from the estate, which, with £16,011 received in dividends, etc., upon securities transferred to the Fund, makes the total income from this source to August 4 £24,011.

The Committee recommend that this sum be added to the collections, making the total of the Fund to August 4 £56,710.

The Fund has received £125 further on account of Mr. C. E. Layton's bequest.

This year 259 institutions have applied for grants from the Fund, being three more than in 1910, and the Committee now recommend the distribution of £56,500 to 166 hospitals and institutions, 59 dispensaries, and 32 nursing associations as shown in the appendix hereto.

The Committee having reduced the bases of award to 29 institutions, the governing bodies of the same were informed thereof in accordance with Law V. Ten deputations attended the Committee, and after hearing their several explanations, the awards were finally determined.

Seven and a half per cent. of the total sum available for distribution is appropriated to the purchase of surgical appliances during the ensuing year, and 2½ per cent. for district nursing associations.

The Comittee have had under consideration the resolution of the Council passed on March 28 last, that in the event of the Fund suffering any diminution which might be fairly attributable to the running of Sunday Cinematograph shows by hospitals, the Committee should take the fact into consideration when recommending their awards for the year, and whilst noticing with regret that there is a reduction in the amount of the Fund compared with the corresponding period of last year, the Committee are not satisfied that such falling off has been caused by the continuance of the shows referred to in the Council's resolution, and therefore refrain from making any recommendation thereon.

The Committee received a deputation from St. George's Hospital, when the questions which have arisen as to the relations between that institution and its medical school were fully discussed. The committee were assured that not one penny of the money subscribed by the Hospital Sunday Fund or from any other source to the general funds of the hospital is used for any purpose whatever save the maintenance and treatment of the patients, and this assurance has since been confirmed by the treasurer in writing. The Committee therefore recommend an award to St. George's Hospital as usual.

In compliance with an order of the Council, statistics have been prepared as usual for the special use of its members, showing the number of beds in hospitals, the cost of patients at hospitals and dispensaries, the proportionate expense of management to maintenance, and other valuable information. A copy thereof has been sent to each member of the Council.

T. VEZEY STRONG, Lord Mayor, President and Treasurer.

JOHN C. BELL. ROBERT GREY.
F. A. BEVAN. G. H. MAKINS.
WILLIAM S. CHURCH. F. H. NORMAN.
SAVILE CROSSLEY. E. PARKER YOUNG.
JOSEPH C. DIMSDALE.

Mansion House, E.C., July 21, 1911.

### Awards Recommended by the Committee of Distribution for the year 1911.

### THIRTY-THREE GENERAL HOSPITALS.

Charing Cross Hospital, £931 17s. 3d.; French Hospital, £300 16s. 2d.; German Hospital, £509 3s. 10d.; Great Northern Central Hospital, £1,078 18s. 2d.; Guy's Hospital, £1,561 4s. 2d.; Hampstead and North-West London Hospitals, £628 10s. 2d.; Italian Hospital, £200 16s. 5d.; Kensington General Hospital, £73 2s. 6d.; King's College Hospital, £1,311 11s. 9d.; London Hospital, £5,037 10s.; London Homœopathic Hospital, £336 2s.; Phillips' Memorial Homœopathic Hospital, £29 8s. 2d.; London Temperance Hospital, £588 3s. 6d.; Metropolitan Hospital, £966 6s. 3d.; Mildmay Hospital, £199 19s. 8d.; Mildmay Memorial Hospital, £33 12s. 2d.; Miller Hospital and Royal Kent Dispensary, £252 1s. 6d.; Poplar Hospital, £535 8s. 9d.; Prince of Wales' General Hospital, Tottenham, £743 12s. 5d.; Royal Free Hospital, £1,214 3s. 8d.; St. George's Hospital, £1,554 9s. 8d.; S S. John and Elizabeth Hospital, £58 16s. 5d.; St. John's Hospital, Lewisham, £180 13s.; St. Mary's Hospital, £1,922 10s. 11d.; St. Thomas's Hospital, £459 12s. 3d.; Seamen's Hospital Society, £1,483 1s. 4d.; the Middlesex Hospital and Convalescent Home, £2,361 3s.; University College Hospital, £1,949 8s. 7d.; Walthamstow, etc., Hospital, £113 8s. 9d.; Wandsworth, Bolingbroke Hospital, £341 19s. 8d.; West Ham Hospital, £495 15s.; West London Hospital, £1,113 7s. 1d.; Westminster Hospital, £1,262 18s. 5d.

### SPECIAL HOSPITALS.

SIX CHEST HOSPITALS.—City of London Hospital for Diseases of the Chest, Victoria Park, £989 0s. 1d.; Hospital for Consumption, Brompton, £2,522 10s. 1d.; Mount Vernon Consumption Hospital, Hampstead, £1,252 16s. 10d.; Royal Hospital for Diseases of the Chest, City Road, £498 5s. 5d.; Royal National Sanatorium (Bournemouth), £84 0s. 6d.; Royal National Hospital for Consumption (Ventnor), £210 1s. 3d.

TWENTY CHILDREN'S HOSPITALS.—Alexandra Hospital for Hip Disease, £273 1s. 8d.; Banstead Surgical Home, £25 4s. 2d.; Barnet Home Hospital, £36 19s. 6d.; Belgrave Hospital for Children, £147 17s. 9d.; Cheyne Hospital for Incurable Children, £94 2s. 2d.; East London Hospital for Children, Shadwell, £757 1s. 3d.; Evelina Hospital for Sick Children, Southwark, £34 2s. 6d.; Home for Incurable Children, Hampstead, £38 13s.; Home for Sick Children, Sydenham, £105 17s. 4d.; Home for Sick Children, Great Ormond Street, £1,134 7s. 2d.; Infants' Hospital, Westminster, £184 17s. 1d.; Kensington, for Children, and Dispensary, £83 3s. 9d.; Paddington Green Hospital for Children, £203 6s. 9d.; Queen's Hospital for Children, Hackney Road, £789 16s. 8d.; Royal Waterloo Hospital for Children and Women, £371 7s. 9d.; St. Mary's Hospital, Plaistow, £217 12s. 9d.; St. Monica's Hospital, Brondesbury, £69 14s. 10d.: Victoria Hospital for Children, Chelsea, £644 9s. 6d.; Victoria Home, Margate, £26 17s. 9d.; Hospital for Hip Disease, Sevenoaks, £54 12s. 3d.

EIGHT LYING-IN HOSPITALS.—British Lying-in Hospital, Endell Street, £121; City of London Lying-in Hospital, City Road, £159 13s.; Clapham Maternity Hospital and Dispensary, £35 5s. 10d.; East End Mothers' Home, £64 14s. 1d.; General Lying-in Hospital, Lambeth, £161 6s. 7d.; Plaistow Maternity Hospital, £41 3s. 4d.; Queen Charlotte's Lying-in Hospital, Marylebone Road, £470 10s. 10d.; Home for Mothers and Babies, Woolwich, £47 1s. 1d.

FIVE HOSPITALS FOR WOMEN.—Chelsea Hospital for Women, £277 5s. 8d.; Hospital for Women, Soho Square, £243 13s. 6d.; Grosvenor Hospital for Women and Children, Vincent Square, £132 15s. 3d.; New Hospital for Women, Euston Road, £273 1s. 7d.; Samaritan Free Hospital, Marylebone Road, £352 18s. 1d.

### TWENTY-THREE OTHER SPECIAL HOSPITALS.

Cancer Hospital, Brompton, Nil; Middlesex Hospital, Cancer Wing, £189 17s. 11d.; London Fever Hospital, Islington, £252 1s. 6d.; Gordon Hospital for Fistula,

Vauxhall Bridge Road, £31 1s. 10d.; St. Mark's Hospital for Fistula, City Road, £271 8s.; National Hospital for the Diseases of the Heart, Soho Square, £136 19s. 3d.; Female Lock Hospital, Harrow Road, £220 2s. 11d.; Hospital for Epilepsy, Paralysis and other diseases of the Nervous System, Maida Vale, £176 9s.; National Hospital for the Paralysed and Epileptic, £816 14s. 6d.; West End Hospital for Diseases of the Nervous System, £358 6s. 3d.; Central London Ophthalmic Hospital, Gray's Inn Road, £134 8s. 10d.; Royal Eye Hospital, St. George's Circus, £289 0s. 11d.; Royal London Ophthalmic Hospital, City Road, £841 2s. 3d.; Royal Westminster Ophthalmic Hospital, Charing Cross, £105 17s. 6d.; Western Ophthalmic Hospital, Marylebone Road, £95 15s. 9d.; Royal National Orthopædic Hospital, Great Portland Street, £410 0s. 11d.; Royal Sea Bathing Hospital, Margate, £138 12s. 11d.; St. John's Hospital for Diseases of the Skin, £63 0s. 5d.; St. Peter's Hospital for Stone, Covent Garden, £48 14s. 8d.; Central London Throat and Ear Hospital, Gray's Inn Road, £51 5s. 1d.; London Throat Hospital, Great Portland Street, £26 0s. 11d.; Royal Ear Hospital, Dean Street, £36 19s. 5d.; Royal Dental Hospital of London, £277 5s. 8d.; National Dental Hospital, 149 Great Portland Street, £43 13s. 9d.

### THIRTY-FOUR CONVALESCENT HOSPITALS.

Metropolitan Convalescent Institution, Walton, £326 0s. 5d.; Metropolitan Convalescent Institution, Broadstairs, £214 5s. 3d.; Metropolitan Convalescent Institution, Bexhill, £398 5s. 8d.; All Saints' Convalescent Hospital, Eastbourne, £336 2s.; All Saints' Convalescent Home, St. Leonards-on-Sea, £21 0s. 1d.; Ascot Priory Convalescent Home, £71 8s. 5d.; Brentwood Convalescent Home for Children, £10 1s. 8d.; Charing Cross Hospital Convalescent Home, Limpsfield, £57 19s. 8d.; Chelsea Hospital for Women Convalescent Home, St. Leonards, £49 11s. 7d.; Metropolitan Hospital Home, Cranbrook, Nil; Deptford Medical Mission Convalescent Home, Bexhill, £17 12s. 10d.; Mrs. Gladstone's Convalescent Home, Mitcham, £65 10s. 10d.; Friendly Societies' Convalescent Home, Dover, £84 0s. 6d.; Hahnemann Convalescent Home, Bournemouth, £25 4s. 2d.; Hanwell Convalescent Home, £10 1s. 8d.; Hastings, Fairlight Convalescent Home, £32 15s. 5d.; Hendon, Ossulston Home, £48 14s. 9d.; Herbert Convalescent Home, Bournemouth, £16 16s. 1d.; Herne Bay Baldwin-Brown Convalescent Home, £50 8s. 4d.; Homœopathic Hospital Convalescent Home, Eastbourne, £6 14s. 6d.; Hemel Hempstead Convalescent Home, £21 0s. 1d.; Mrs. Kitto's Convalescent Home, Reigate, £42 0s. 2d.; London Hospital Convalescent Home, Tankerton, £58 16s. 5d.; Mary Wardell Convalescent Home for Scarlet Fever, £50 8s. 4d.; Police Seaside Home, Brighton, £47 17s. 10d.; St. Andrew's Convalescent Home, Clewer, £84 0s. 4d.; St. Andrew's Convalescent Home, Folkestone, £151 5s.; St. John's Home for Convalescent and Crippled Children, Brighton, £30 4s. 10d.; St. Joseph's Convalescent Home, Bournemouth, £35 5s. 9d.; St. Leonards-on-Sea Convalescent Home for Poor Children, £84 0s. 4d.; Eversfield, St. Leonards, £25 4s. 2d.; St. Mary's Convalescent Home, Broadstairs, £126 0s. 9d.; St. Mary's Convalescent Home, Shortlands, £15 9s. 3d.; St. Michael's Convalescent Home, Westgate-on-Sea, £29 8s. 1d.; Seaside Convalescent Hospital, Seaford, £75 12s. 6d.

### TWENTY-FOUR COTTAGE HOSPITALS.

Acton Cottage Hospital, £101 12s. 10d.; Beckenham Cottage Hospital, £67 4s. 5d.; Blackheath and Charlton Cottage Hospital, £73 18s. 11d.; Bromley, Kent, Cottage Hospital, £101 12s. 10d.; Bushey Heath Cottage Hospital, £31 1s. 9d.; Canning Town Cottage Hospital, £77 6s. 1d.; Chislehurst, Sidcup and Cray Valley Cottage Hospital, £58 16s. 5d.; Coldash Cottage Hospital, £21 0s. 1d.; Ealing, £74 15s. 9d.; East Ham Cottage Hospital, £59 13s. 2d.; Eltham Cottage Hospital, £63 0s. 5d.; Enfield Cottage Hospital, £73 18s. 11d.; Epsom and Ewell Cottage Hospital, £36 19s. 5d.; Hounslow Cottage Hospital, £39 9s. 9d.; Livingstone Dartford Cottage Hospital, £75 12s. 6d.; Kingston, Victoria Hospital, £64 14s.; Reigate and Redhill Hospital, £110 1s. 5d.; Sidcup Cottage Hospital, £29 8s. 2d.; Teddington, £85 14s. 1d.; Tilbury (Passmore Edwards) Cottage Hospital, £42 0s. 2d.; Willesden Cottage Hospital, £92 8s. 6d.; Wimbledon Cottage Hospital, £57 2s. 10d.; Wimbledon (South) Cottage Hospital, £42 0s. 2d.; Wood Green Cottage Hospital, £62 3s. 8d.

### THIRTEEN INSTITUTIONS.

Hospital for Invalid Gentlewomen, Lisson Grove, £74 15s. 9d.; St. Saviour's Hospital and Nursing Home, £79 16s. 6d.; Invalid Asylum, Stoke Newington, £27 14s. 7d.; Firs Home, Bournemouth, £31 18s. 6d.; St. Catherine's Home, Ventnor, £16 16s. 1d.; Friedenheim Hospital for the Dying, £207 10s. 9d.; Free Home for Dying, Clapham, £121 16s. 9d.; St. Luke's House, Pembridge Square, £134 8s. 9d.; Santa Claus Home, Highgate, £49 11s. 7d.; Royal Mineral Water Hospital, Bath, £50 8s. 4d.; Winifred House, Holloway, £52 18s. 7d.; All Saints, Highgate, £28 11s. 5d.; Erskine House, Hampstead, £26 17s. 9d.

### FIFTY-NINE DISPENSARIES.

Battersea Provident Dispensary, £176 9s.; Billingsgate Dispensary, £39 9s. 9d.; Blackfriars Provident Dispensary, £15 19s. 4d.; Bloomsbury Provident Dispensary, £13 8s. 10d.; Brixton, etc., Dispensary, £40 6s. 7d.; Brompton Provident Dispensary, £16 16s. 1d.; Buxton Street Dispensary, £13 8s. 10d.; Camberwell Provident Dispensary, £63 17s. 2d.; Camden Town Provident Dispensary, £12 12s. 1d.; Chelsea, Brompton and Belgrave Dispensary, £32 15s. 5d.; Chelsea Provident Dispensary, £11 15s. 3d.; Childs Hill Provident Dispensary, £10 1s. 8d.; City Dispensary, £59 13s. 2d.; Clapham General and Provident Dispensary, £21 0s. 1d.; Deptford Medical Mission, £21 0s. 1d.; Eastern Dispensary, £36 2s. 7d.; East Dulwich Provident Dispensary, £51 5s. 1d.; Farringdon General Dispensary, £38 13s.; Finsbury Dispensary, £36 19s. 5d.; Forest Hill Provident Dispensary, £31 1s. 9d.; Greenwich Provident Dispensary, £25 4s. 2d.; Hackney Provident Dispensary, £19 6s. 4d.; Hampstead Provident Dispensary, £46 4s. 2d.; Holloway and North Islington Dispensary, £8 8s.; Islington Dispensary, £43 13s. 10d.; Islington Medical Mission, £36 19s. 5d.; Kennington and Vauxhall Provident Dispensary, £11 15s. 3d.; Kensal Town Provident Dispensary, £10 1s. 8d.; Kentish Town Medical Mission, £19 6s. 4d.; Kilburn, Maida Vale, and St. John's Wood Dispensary, £30 5s.; Kilburn Provident Medical Institution, £42 0s. 2d.; London Dispensary, Spitalfields, £14 5s. 7d.; London Medical Mission, Endell Street, £84 0s. 4d.; Margaret Street, for Consumption, £28 11s. 6d.; Metropolitan Dispensary, £49 11s. 7d.; Mildmay Medical Mission Dispensary, £9 4s. 9d.; Notting Hill Provident Dispensary, £15 19s. 3d.; Paddington Provident Dispensary, £30 5s.; Public Dispensary, Drury Lane, W.C., £31 18s. 6d.; Queen Adelaide's Dispensary, £21 16s. 10d.; Royal General Dispensary, £26 0s. 11d.; Royal Pimlico Provident Dispensary, £32 15s. 5d.; Royal South London Dispensary, £38 13s.; St. George's, Hanover Square,

Dispensary, £27 14s. 7d.; St. John's Wood Provident Dispensary, £38 13s.; St. Marylebone General Dispensary, £40 6s. 6d.; St. Pancras and Northern Dispensary, £32 18s. 6d.; South Lambeth, Stockwell, and North Brixton Dispensary, £26 17s. 8d.; Stamford Hill, Stoke Newington Dispensary, £45 7s. 6d.; Tower Hamlets Dispensary, £34 8s. 11d.; Walworth Provident Dispensary, £12 12s.; Wandsworth Common Provident Dispensary, £10 1s. 8d.; Westbourne Provident Dispensary, £14 5s. 7d.; Western Dispensary, £67 4s. 5d.; Western General Dispensary, £78 2s. 11d.; West Ham Provident Dispensary, £9 4s. 9d.; Westminster General Dispensary, £38 13s.; Whitechapel Provident Dispensary, £29 8s. 3d.; Woolwich Provident Dispensary, £34 8s. 11d.

THIRTY-TWO NURSING ASSOCIATIONS.

Belvedere, Abbey Wood, £6; Brixton, £18; Central St. Pancras, £18; Chelsea and Pimlico, £18; Hackney, £24; Hammersmith, £42; Hampstead, £18; Isleworth, £12; Kensington, £42; Kilburn, £6; Kingston, £30; Lambeth Road (Catholic), £12; Metropolitan (Bloomsbury), £12; St. Olave's (Bermondsey), £18; Paddington and Marylebone, £30; Peckham, £12; Plaistow, £120; Plaistow (Maternity), £156; Rotherhithe, £12; Shoreditch, £54; Sick Room Helps Society, £18; Sidcup, £6; Silvertown, £18; South London (Battersea), £54; Southwark, £42; South Wimbledon, £42; Tottenham, £6; Westminster, £24; Woolwich, £36; East London, £150; North London, £60; London District, £300.

